# Feasibility of urinary microRNA detection in breast cancer patients and its potential as an innovative non-invasive biomarker

**DOI:** 10.1186/s12885-015-1190-4

**Published:** 2015-03-28

**Authors:** Thalia Erbes, Marc Hirschfeld, Gerta Rücker, Markus Jaeger, Jasmin Boas, Severine Iborra, Sebastian Mayer, Gerald Gitsch, Elmar Stickeler

**Affiliations:** 1 Department of Obstetrics and Gynecology, University Medical Center Freiburg, Hugstetterstr. 55, Freiburg, 79106 Germany; 2 German Cancer Consortium (DKTK), Heidelberg, Germany; 3 German Cancer Research Center (DKFZ), Heidelberg, Germany; 4 Institute for Medical Biometry and Statistics, University Medical Center Freiburg, Freiburg, Germany

**Keywords:** Breast cancer, microRNA, Urine, Biomarker, Non-invasive, Innovative, Discrimination

## Abstract

**Background:**

Since recent studies revealed the feasibility to detect blood-based microRNAs (miRNAs, miRs) in breast cancer (BC) patients a new field has been opened for circulating miRNAs as potential biomarkers in BC. In this pilot study, we evaluated to our knowledge for the first time whether distinct pattern of urinary miRNAs might be also applicable as innovative biomarkers for BC detection.

**Methods:**

Urinary miRNA expression levels of nine BC-related miRNAs (miR-21, miR-34a, miR-125b, miR-155, miR-195, miR-200b, miR-200c, miR-375, miR-451) from 24 untreated, primary BC patients and 24 healthy controls were quantified by realtime-PCR. The receiver operating characteristic analyses (ROC) and logistic regression were calculated to assess discriminatory accuracy.

**Results:**

Significant differences were found in the expression of four BC-associated miRNAs quantified as median miRNA expression levels. Urinary miR-155 levels were significantly higher in BC patients compared to healthy controls (1.49vs.0.25; p < 0.001). In contrast, compared to healthy controls, BC patients exhibited significantly lower urinary expression levels of miR-21 (2.27vs.5.07; p < 0.001), miR-125b (0.71vs.1.62; p < 0.001), and miR-451 (0.02vs.0.59 p = 0.004), respectively. The ROC including all miRNAs as well as the group of the four significant deregulated miRNAs separated BC patients from healthy controls with a very high (area under the receiver operating characteristic curve [AUC] = 0.932) and high accuracy (AUC = 0.887), respectively.

**Conclusions:**

We were able to demonstrate for the first time the feasibility to detect distinct BC-dependent urinary miRNA profiles. The expression levels of four urinary miRNAs were specifically altered in our cohort of BC patients compared to healthy controls. This distinct pattern offers the possibility for a specific discrimination between healthy women and primary BC patients. This sustains the potential role of urinary miRNAs as non-invasive innovative urine-based biomarkers for BC detection.

**Electronic supplementary material:**

The online version of this article (doi:10.1186/s12885-015-1190-4) contains supplementary material, which is available to authorized users.

## Background

Small non-coding microRNAs (miRNAs, miRs) with a length of approximately 22 nucleotides are important post-transcriptional regulators of numerous human genes. MiRNAs modulate the expression of tumor suppressor genes as well as oncogenes [1-3]. In breast cancer (BC), emerging evidence suggests a potential role for deregulated miRNAs as modulators of carcinogenesis, proliferation, apoptosis and drug-resistance, respectively [4]. Most data exist for tumor tissue or breast cancer cell line-based miRNA expression profiles [5,6]. However, there are numerous hypotheses for a pivotal role of miRNAs in intercellular communication [7,8] partially based on the leakage of miRNAs in circulation [9] as well as by active and passive export mechanisms, respectively [9]. Recent studies documented the feasibility to detect stable miRNAs in serum and plasma. This opened the field for these circulating miRNAs as potential novel biomarkers in BC for early detection but also outcome prediction [10-13]. Our extensive literature research revealed the following nine miRNAs as actually relevant in BC, especially as potential blood based biomarker in discrimination BC from healthy controls or as predictors in therapy response (Table 1). For example, high expression serum levels of miR-10b, 34a and 155 were associated with primary metastatic BC (p < 0.05) and high miR-34a levels correlated with an advanced stage of disease (p = 0.01) [13]. Additional data revealed a strong correlation between serum miR-122 and miR-375 levels and neoadjuvant chemotherapy response in locally advanced BC [14]. Overexpression of miR-21 in BC tissue as well in blood based studies has a relevant oncogenic role by promoting invasion, proliferation and metastases and poor prognosis in BC patients [10,15,16]. Emerged studies showed up-regulated miR-125b serum levels in BC patients as an innovative serum biomarker for discrimination BC patients from healthy controls and to predict chemotherapeutic resistance [17,18]. Other studies indicated miR-155 and miR-195 as promising diagnostic targets, while miR-155 is also discussed as a potential therapeutic target in BC [12,19-22]. The role of miR-200 family in blocking tumor angiogenesis by inhibition epithelial-mesenchymal transition represents a potential relevant therapeutic predictive parameter in BC therapy [17,23]. Most interestingly, in one study higher expression levels of miR-200b and miR-200c were observed in serum from circulating tumor cells (CTC)-positive metastatic BC patients compared to CTC-negative patients and promised miR-200b and miR-200c as an indicator for CTC-status and a prognostic marker in metastatic BC [18]. In regard of BC detection and discrimination from healthy controls miR-451 in combination with miR-145 were identified as the best potential circulating biomarker [24].

**Table 1:** **Functional implications of circulating microRNAs and their characteristic features in breast cancer patients**

miRNA	signaling pathways	target genes	source	characteristic BC features	references
**miR-21**	apoptosis; EGFR	PDCD4, PTEN, BCL-2, HER2, FAS, TPM1	serum	↗ in primary BC, correlation to tumor size and lymph node status	[10,15,16,25-29]
**miR-34 a**	vascularization; EGFR, β-Catenin	VEGF, MYC, BCL2, WNT, p53	serum	↗ in metastatic BC compared to primary BC and controls	[13,30]
**miR-125b**	apoptosis; EGFR	HER2, p53, BAK1	serum	↗ in primary BC, prediction of chemotherapy resistance	[17,18,31-33]
**miR-155**	Akt; apoptosis; morphogenesis; EMT	VHL, VEGF, p53, TGF- β	serum	↗ in primary BC; ↘ after surgery and chemotherapy	[20,21,34,35]
**miR-195**	apoptosis	BCL 2, CDK6	whole blood	↗ in primary BC	[12,19]
			serum	↘ in primary BC	[22]
**miR-200b**	EMT	ZEB1/2, E-Cadherin	plasma	↗ in metastatic BC; correlation to CTC status	[18,36]
**miR-200c**	EMT	ZEB1/2, E-Cadherin	plasma	↗ in metastatic BC; correlation to CTC status	[18,37]
**miR-375**	apoptosis	14-3-3 Protein	plasma	↗ in metastatic BC	[14,18]
**miR-451**	MDR	MDR1, 14-3-3 Protein	plasma, serum	↗ in primary BC	[24,38,39]

MiRNA specimen pre-selection for this study was based on previous investigations elucidating functional features and interrelations of miRNA expression in regard to breast carcinogenesis.

*↗: increased, ↘: decreased expression levels of miRNAs in comparison to healthy controls; EMT: epithelial-mesenchymal-transition; CTC: circulating tumor cells; MDR: multi-drug resistance.*

So far, urine, as an easy approachable compartment and a non-invasive source for circulating miRNAs, has not been tested in the setting of BC while current studies suggest a high potential of urinary miRNAs in urologic cancers [10]. In this pilot study, we evaluated to our knowledge for the first time whether circulating urinary miRNA pattern might be applicable as potential biomarkers for BC detection. Therefore we assessed the expression of a distinct panel of BC associated miRNAs (miR-21, miR-34a, miR-125b, miR-155, miR-195, miR-200b, miR-200c, miR-375, miR-451, respectively) in female healthy controls in comparison to newly diagnosed, so far untreated BC patients.

## Methods

### Cohorts and sampling

Midstream specimen of urine (MSU) were collected in a case–control cohort of 24 untreated patients, newly diagnosed with primary BC in the adjuvant setting and of 24 healthy female controls at the Department of Obstetrics and Gynecology, University Medical Center Freiburg during September 2011 to August 2012. Exemplarily, serum samples of four consecutive patients and healthy controls were collected for a comparative analysis with corresponding urine specimen. The specimen of urine and serum were collected from healthy women confirmed not to have BC and no history of other (malignant) diseases or current inflammation. For all BC patients, distant metastasis was excluded by staging procedures according to the current national guidelines. The institutional ethical review board of the University of Freiburg, approved the investigation protocol (36/12). All patients and healthy controls involved, gave written informed consent for participation in this study. In Table 2 the characteristics of the study population are summarized. All MSU specimen were centrifuged extensively to eradicate contamination with any urothelial or microbiological cell material. Supernatant was used for subsequent analysis. Samples were stored at −80°C until further processing.

**Table 2:** **Characteristics of breast cancer (BC) patients and healthy controls**

	BC patients	healthy controls	p value
N	24	24	
Median age, y	54	52	0.070
**Histology**			
Invasive ductal	22		
Invasive lobular	2		
**Tumor stage**			
pT1	13		
pT2	8		
pT3	3		
**Nodal status**			
pN0	15		
pN1	5		
pN2	4		
**Grading**			
G1	2		
G2	13		
G3	9		
**Hormone receptor status**			
ER positive	22		
PR positive	20		
**HER2neu status**			
Positive	2		
**Mastectomy**			
Yes	4		
No	20		

Relevant characteristics of 24 BC patients and 24 healthy controls are demonstrated.

### Statistical analysis

The statistical analyses were performed by using the SPSS software package, version 22.0 (SPSS Inc. Chicago, IL, USA) and the open available statistical software environment R (R Development Core Team, “R: A Language and Environment for Statistical Computing”. R foundation for Statistical Computing, 2013. URL http://www.R-project.org). Mann Whitney-U test was applied to test the median urinary expression levels of miR-21, miR-34a, miR-125b, miR-155, miR-195, miR-200b, miR-200c, miR-375, and miR-451, respectively. Logistic regression was used to combine all miRNAs to a score which is interpreted as a diagnostic marker for discrimination of cases and controls. Its accuracy was investigated by an ROC (receiver operating characteristic) curve, the area under the curve (AUC) and accuracy measures for a suitable cut-off value.

### RNA isolation

Norgen’s Urine microRNA Purification Kit (#29000, Norgen Biotek Corporation, Thorold, ON, Canada) was applied for isolation and purification of small RNA molecules (< 200 nt). According to the manufacturer’s protocol 1 ml urine per sample was lysed and RNA was isolated and purified in a spin column procedure. Serum samples were diluted 1:1 with water (RNAse-free, DEPC treated) to lower protein load before parallel RNA isolation with Norgen’s kit. Purified miRNA was finally collected in 50 μl RNA Elution buffer (Kit component) and RNA concentration determined densitometrically using Eppendorf Biophotometer (Eppendorf, Hamburg, Germany). All miRNA samples were stored at −80°C.

### Reverse transcription

Generation of miRNA-cDNA was performed by Reverse Transcription of 250 ng miRNA/sample applying Megaplex™ Primer Pools, Human Pools A v2.1 (#4401009, Applied Biosystems®, Life Technologies™, Thermo Fischer Scientific Inc., Schwerte, Germany) in a total reaction volume of 20 μl. cDNA probes were stored at 4°C.

### Pre-amplification

Enhancement of miRNA-cDNA quantity was achieved by application of Megaplex™ PreAmp Primers, Human Pool A (#4399233, Applied Biosystems®). Thereto 5 μl of miRNA-cDNA generated by Reverse Transcription were pre-amplified in a 20 μl reaction mix according to the manufacturer’s protocol. Following pre-amplification, miRNA-cDNA probes were diluted in RNAse free water (1:3, final volume 60 μl) for subsequent PCR analysis and stored at 4°C.

### Quantitative realtime-PCR

MiRNA expression levels were determined by quantitative realtime-PCR applying TaqMan® MicroRNA Assays (#4427975, Applied Biosystems®). 1 μl miRNA-cDNA per sample was used in a total reaction volume of 10 μl according to the manufacturer’s protocol on Mastercycler® ep Realplex (Eppendorf AG, Hamburg, Germany). Relative quantification of different miRNA types resulted from ΔC_t_ method normalized on corresponding median expression values of housekeeping miRNAs miR-16 and miR-26b. Data acquisition is based upon mean values of duplicate PCR analysis.

## Results

As an essential prior condition for reliable miRNA quantification analysis in urine, the expression levels of various miRNA types were investigated in regard to their potential role as solid housekeeping genes (HKG) in this clinical study. Since robust housekeepers of tissue-based miRNA analyses (e.g. snRNA U) had to be excluded in advance, our preliminary qPCR-based scanning procedure could identify miR-16 and mir-26b as potential candidates. Among the potential HKGs within the range offered by supplier (ABI), expression data analysis was performed applying ‘BestKeeper’, an Excel-based tool using pair-wise correlations for the determination of stable housekeeping genes, differentially regulated target genes and sample integrity [40]. The assays and subsequent data analysis demonstrated that miR-16 and miR-26b were characterized by stable and consistent expression values in a set of >50 urine specimen – independent of origin from BC patients or healthy controls (BestKeeper; miR-16: p = 0.001; miR-26b: p = 0.001). These results indicate miR-16 and miR-26b in urine as the best internal control for normalization in this experimental approach.

These two miRNAs were already implemented as HKG in different contexts of miRNA expression analyses [10,13,41,42]. In fact, Davoren and colleagues could identify miR-16 and miR-26b as highly ranked suitable housekeeping miRNAs with expression stability calculated from intra- and intergroup variation (NormFinder) and also based on an estimate of pairwise variation (geNorm) [42]. According to current methodological standard procedure in qPCR quantification [43,44] the geometric mean of miR-16 and miR-26b expression served as comparative value for quantitative assessment of relevant miRNAs in a duplicate analysis.

The complete panel of the selected nine miRNAs was detectable in urine by our newly designed qRT-PCR protocol. The findings were reproducible with acceptable inter- and intra-assay variations. Intra-assay standard deviation of corresponding single values in miRNA expression level quantification remained within a range of <0.2%, inter-assay standard deviation within a range of <0.3% (Additional file 1: Figure S1A, B). Expression stability of HKG miR-16 and -26b was determined for both, BC patients and healthy controls (Additional file 2: Figure S2).

**Figure 1:**
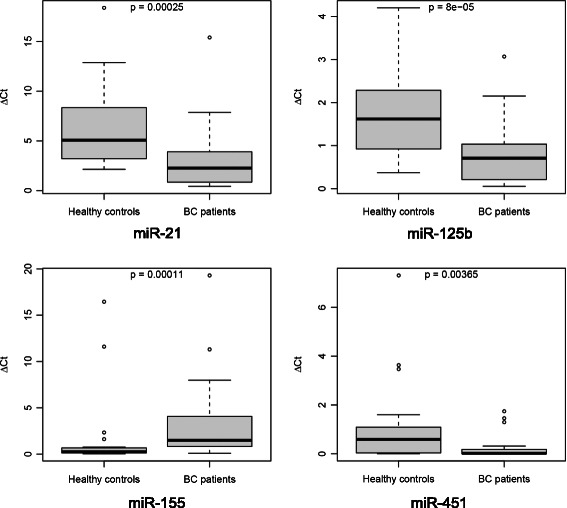
**Box plots of ΔCt-values of significant urinary miRNAs in breast cancer patients compared to healthy controls.** Median urinary expression levels of miR-21 (2.27vs.5.07; p < 0.001), miR-125b (0.72vs.1.62; p < 0.001), and miR-451 (0.02vs.0.590; p = 0.004) were significantly decreased in BC patients compared to healthy controls, respectively. Urinary miRNA-155 expression was significantly increased in BC patients compared to healthy controls (1.49vs.0.25; p < 0.001). Median ΔCt-value and interquartile range of duplicate experiments. Thick lines: median (50% percentile); gray boxes: 25% to 75% percentile; thin lines: minimal and maximal value,^0^: moderate outlier. Mann Withney-U test. Quantitative realtime-PCR.

**Figure 2:**
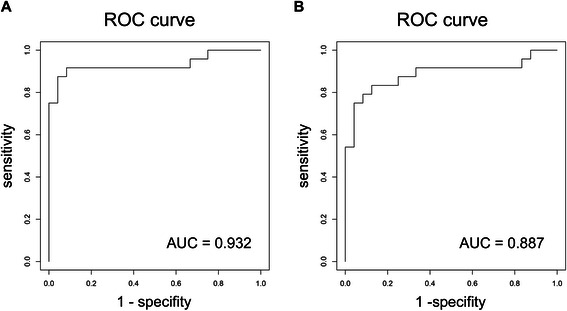
**ROC (receiver operating characteristic) curve of combined miRNA analysis. (A)** ROC curve of all miRNAs for the score combined from all miRNA (miR-21, miR-34a, miR-125b, miR-155, miR-195, miR-200b, miR-200c, miR-375, miR-451) in discrimination between BC patients and healthy controls. A combined ROC (receiver operating characteristic) curve of all miRNAs showed the excellent AUC (area under the curve) of 0.932 and an optimal sensitivity of 0.917 (95%-CI [0.812; 1.000]) and specifity of 0.917 (95%-CI [0.686; 0.978]), respectively. **(B)** ROC curve of the four significantly deregulated miRNAs ( miR-21, miR-125b, miR-155, miR-451) was performed and showed high diagnostic accuracy with an AUC of 0.887 and a sensitivity of 0.833 ( 95%-CI [0.697; 0.997]) and specifity of 0.875 (95%-CI [0.640; 0.957]), respectively.

**Figure 3:**
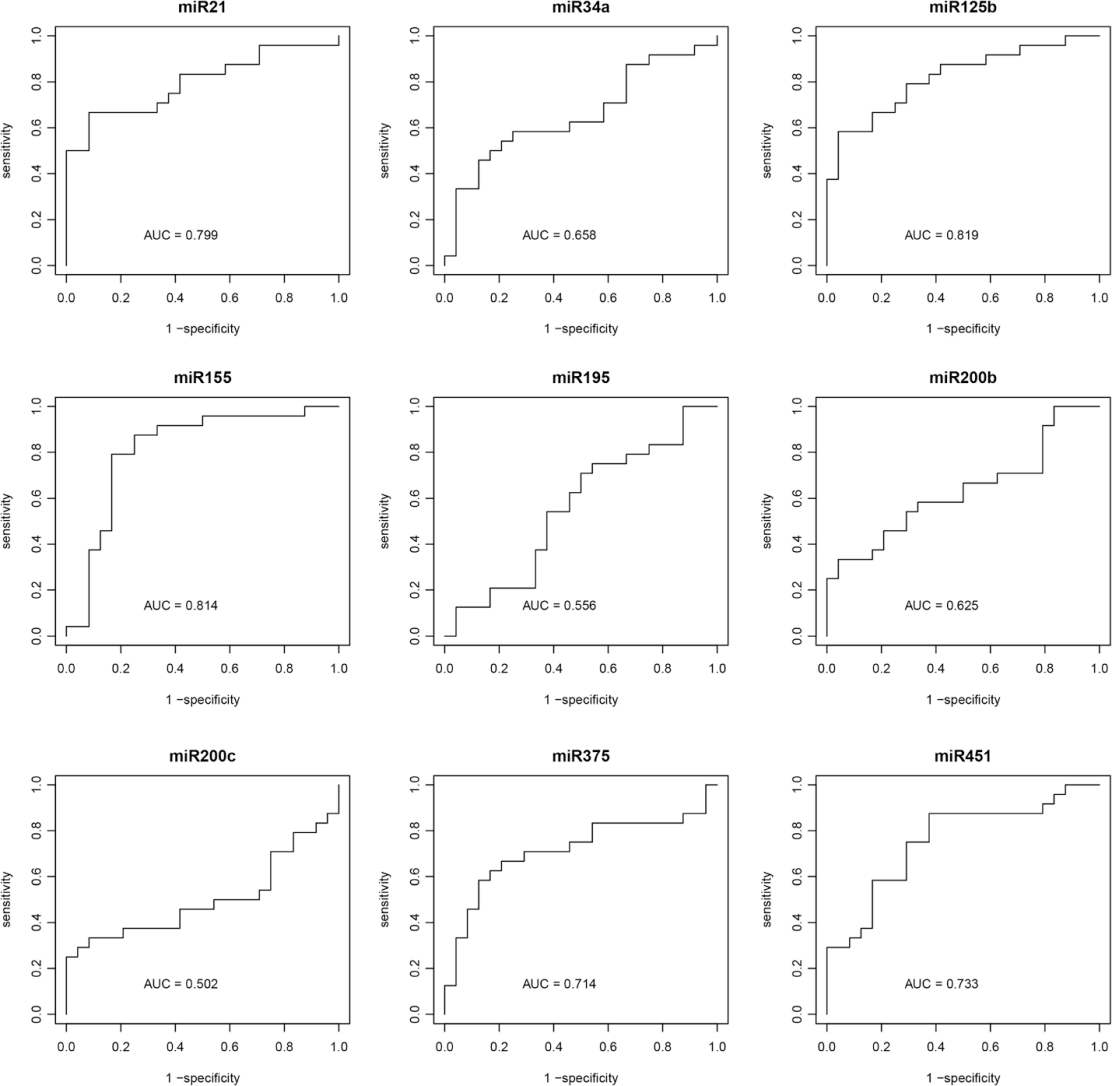
**ROC curves of the diagnostic potential of the individual urinary miRNAs (miR-21, miR-34a, miR-125b, miR-155, miR-195, miR-200b, miR-200c, miR-375, miR-451) in discrimination between BC patients and healthy controls.** The AUC values ranged from 0.502 to 0.819, respectively.

The quantification of urinary expression levels of these miRNAs revealed distinct pattern for both, healthy controls and BC patients, respectively. Our data showed significant differences in the expression of four BC associated miRNAs determined as median ΔC_t_ values of the distinct miRNA specimen normalized against the geometric mean of the two housekeepers miR-16 and miR-26b, respectively. In detail, urinary miRNA-155 expression was significantly increased in BC patients compared to healthy controls (1.49vs.0.25; p < 0.001) (Additional file 3: Table S1; Figure 1). In contrast, compared to healthy controls, BC patients exhibited significantly lower median urinary expression levels of miR-21, (2.27vs.5.07; p < 0.001), miR-125b (0.71vs.1.62; p < 0.001), and miR-451 (0.02vs.0.59; p = 0.004) (Additional file 3: Table S1; Figure 1), respectively. For the additional miRNAs, miR-34a, 195, 200b, 200c, respectively, urinary expression levels did not show any significant differences between BC patients and healthy controls (Additional file 3: Table S1; Additional file 4: Figure S3). MiR-375 demonstrated a strong tendency towards significant expression differences between BC patients group vs. controls (4.56vs.9.29; p = 0.011) (Additional file 3: Table S1; Additional file 4: Figure S3). ROC curve analyses were performed to evaluate the diagnostic power of the selected urinary miRNAs for BC detection. The combined nine miRNAs revealed with an excellent AUC of 0.932, an optimal sensitivity of 0.917 (95%-CI [0.812; 1.000]) as well as specifity of 0.917 (95%-CI [0.686; 0.978]), respectively, the best diagnostic accuracy in discrimination of BC patients from healthy controls (Figure 2A). A scoring approach employing only the four significantly altered miRNAs (miR-21, miR-125b, miR-155 and miR-451) still revealed a good but lower diagnostic accuracy when compared to the nine miRNA score, with an AUC of 0.887, sensitivity of 0.833 (95%-CI[0.697; 0.997]) and specifity of 0.875 (95%-CI [0.640; 0.957]), respectively (Figure 2B). In contrast, the accuracy dropped significantly, when the four latter mentioned miRNAs were solitarily analyzed with an AUC ranging from 0.819 to 0.773 (Figure 3).

The comparative subsequent analysis of these miRNA profiles in serum of BC patients (n = 4) and healthy controls (n = 4) showed no significant differences in median serum levels between the two groups, respectively. In addition the intra-group comparison of urinary to serum miRNA levels in BC patients as well as in healthy controls demonstrated no interrelation between the two different compartments (Additional file 3: Tables S2–S4). Interestingly, all urine samples tested were characterized by miR-375 expression, while corresponding serum samples did not show any detectable miR-375 levels.

## Discussion

There is a growing body of evidence for a role of circulating miRNAs in the serum and plasma of BC patients as a potential non-invasive biomarker. However, data regarding miRNAs in urine, as an extracellular fluid compartment, are not available for BC patients. Our pilot study proofs to our knowledge for the first time the possibility to detect BC related miRNA levels in urine and to use specific urine miRNA pattern as biomarker for BC. In urine of healthy controls and patients, newly diagnosed for BC, we analyzed a panel of nine BC associated miRNAs (miR-21, miR-34a, miR-125b, miR-155, miR-195, miR-200b, miR-200c, miR-375, miR-451, respectively). We were able to demonstrate that the expression levels of four urinary miRNAs were specifically and significantly altered in our cohort of 24 breast cancer patients. Furthermore, ROC analyses demonstrated a significant improvement of the diagnostic potential and accuracy when the nine investigated miRNAs were combined. For this miRNA panel we were able to reach a discriminatory power of AUC = 0.932. Even scoring with the four most altered miRNAs (miR-21, miR-125b, miR-155, miR-451) the accuracy was high with an AUC of 0.887. Urine levels of miR-155 were significantly induced in BC when compared to healthy controls. These findings are in line with recently published studies, which reported an overexpression of miR-155 in sera and tissue samples of primary BC patients [31,33]. MiR-155 acts as a multifunctional miRNA with important roles in several physiological and pathological processes such as inflammation, immunity, cancer and cardiovascular disease, respectively, and was already discussed as a potential blood-based biomarker [31,45] . Most interestingly, the high urinary levels of miR-155 are strongly supported by previous studies pointing out a clear clinical correlation of miR-155 expression and breast malignancies [20,21]. High serum levels of miR-155 were described in BC patients before surgery or chemotherapy, while both treatment options significantly reduced levels of circulating miR-155 in serum [21]. The functional and clinical knowledge on miR-155 clearly summarizes its oncogenic role in breast cancer as reviewed by Mattiske et al. [20].

In contrast, the other specifically regulated urinary miRNAs (miR-21, miR-125b, miR-375 and miR-451, respectively) displayed significant decreased expression levels compared to healthy controls. These findings are not consistent to the current literature regarding the tissue and blood expression levels of these certain miRNAs. Overexpression of miR-21 in tissue as well as in serum has been correlated to advanced tumor stage, lymph node metastasis and poor prognosis in BC patients [10,16,28,46,47]. It targets the tumor suppressor genes PTEN, Tropomyosin alpha-1 chain (TPM1) and Programmed Cell Death 4 (PDCD4), thereby exhibiting oncogenic activity by promoting tumor cell proliferation and inhibition of apoptosis [25,29]. The differentially expressed miR-125b was found to be up-regulated in sera of BC patients and to have predictive power for chemotherapeutic resistance [31,33], which might be due to a direct interaction of this miRNA with the tumor suppressor p53 and the pro-apoptotic Bcl-2 antagonist killer1 (Bak1) [33]. Emerging evidence suggests miR-375 as a diagnostic as well as a prognostic marker for metastatic breast cancer. High plasma expression levels of miR-375 were found to be a sensitive marker for minimal residual disease with circulating tumor cells and specifically discriminate between metastatic BC patients and healthy controls [18,48]. An additional trial identified high serum levels of miR-375 in combination with miR-122 as positive predictive markers for the response to neoadjuvant chemotherapy in locally advanced BC patients [14].

Induced levels of miR-451 together with miR-145 displayed also potential impact as a diagnostic biomarkers in BC [24]. MiR-451 participates in activation of MDR1/P-glycoprotein expression with an up-regulation in multidrug resistant cancer cell lines [49].

The observed decreased urine levels of the latter miRNAs do not necessarily reflect a contradiction to the known induction in serum and tumor tissues. First, the specimens were derived from complete separated compartments with unknown underlying regulatory mechanisms. Weber et al. showed striking differences of miRNA expression profiles in different human body fluids within an individual, with the lowest variety of miRNA types detectable in urine [50]. The same study demonstrated alterations in miRNA expression profiles that relate to changes in physiological and/or pathological conditions. Most interestingly, some miRNAs showed higher expression levels in urine compared to serum, hence implicating particular miRNA secretion processes in kidney and/or urothelial compartments [50]. The experimental setup in this study does not distinguish free urinary miRNA molecules from miRNA particles packed in and protected by vesicles (exosomes). However, Cheng et al. could show recently, that Norgen isolation kit offers the highest yield of exosomal miRNAs from urine samples among all commercial suppliers tested [51] . Especially the occurrence of high levels of RNase in the urinary tract, which lead to the total degradation of free RNA types, supports our hypothesis that only exosomal miRNAs remain detectable in urine as the investigated compartment in our study [51-53]. The results of miR-375 might serve in this context as a good example. Notably, our subsequent analysis of matched pairs of serum and urine specimen revealed a discrepancy in miR-375 expression. Clear urinary expression was found in both groups, in contrast this miRNA type was not detectable in serum of both, BC patients and controls. The favorable explanation might be, that miR-375 is secreted most likely by cells of the urinary tract and might therefore be not specific for breast cancer (Additional file 3: Tables S2–S4).

The signaling properties of tumor cell-secreted miRNA-packed exosomes on normal cells have been demonstrated in various functional studies [54-56]. The evidence of a dependence between extracellular (blood-based) and cellular (BC tumor tissue based) miRNA profiles is nearly missing [39]. Moreover, a direct correlation between miRNA expression levels in the two extracellular compartments blood and urine has yet not been clearly demonstrated. The induced level of miR-155 together with decreased levels of four distinct miRNAs and four constant miRNA levels strongly suggest a specific phenomenon with distinct regulatory pattern rather than a general unspecific effect.

As a matter of fact this pilot study clearly accounts for the proof of principle for the applicability of urinary miRNA expression profiles as a potential diagnostic tool in BC management. This study is limited by cohort size and the case control design. Furthermore, a wider investigatory approach on larger cohorts of independent patient population is needed to validate the applied ROC scoring system.

Since we have a distinct and exclusive look on the urinary miRNA profile as a diagnostic and potential prognostic/predictive tool, the observed doubtful discrepancies between the existing data for tumor and serum profiles do not compromise the value of our analysis.

## Conclusions

In conclusion, with this pilot trial we demonstrate for the first time the feasibility to detect a BC dependent miRNA profile in urine. We are able to proof the reliability, reproducibility and robustness of our self-developed assay in the complex compartment of urine. The test enables us to specifically discriminate between healthy women and patients with local breast cancer. We could identify four significantly altered and specifically regulated miRNAs (miR-21, miR-125b, miR-451 and miR-155) in BC patients compared to healthy controls. Our present findings show typical expression patterns in the urine of BC patients. This sustains the potential role of urinary miRNAs as non-invasive innovative biomarkers in detection of BC. Since this pilot study examines only a limited number of samples extended future studies are needed to confirm these observations.

## Additional files

Additional file 1: Figure S1. Inter- and intra-assay variance in qPCR analysis in four assays. **A**. Inter-assay variance of miRNA types miR-16, −21, −26b, −34a, −125b, and −155. **B**. Intra-assay variance of miRNA types miR-16, −21, −26b, −34a, −125b, and −155, showing mean standard deviation (SD) in percentage [%] as vertical-bar diagram with numerical values below.
Additional file 2: Figure S2. HKG expression stability in qPCR analysis. Expression values (geometric mean) of housekeeping miRNAs miR-16 and miR-26b in BC patients (BC) and healthy controls (C). Standard deviation (SD) and numerical values below vertical-bar diagram.
Additional file 3: Table S1. Expression levels of urinary miRNAs of BC patients and healthy controls. Median urinary expression levels of nine breast cancer-related miRNAs in 24 BC patients and 24 healthy controls. *Mann Withney-U test, interquartile range in parentheses.***Table S2.** Comparison of miRNA expression levels in serum of BC patients and controls. **Table S3.** Comparison of miRNA expression levels in serum and in urine of BC patients. **Table S4.** Comparison of miRNA expression levels in serum and in urine of controls.
Additional file 4: Figure S3. Box plots of ΔCt-values of all nine investigated urinary miRNAs in breast cancer patients compared to healthy controls. Median urinary expression levels of miR-21, miR-34a, miR-125b, miR-155, miR-195, miR-200b, miR-200c, miR-375, and miR-451. Median ΔCt-value and interquartile range of duplicate experiments. Thick lines: median (50% percentile); gray boxes: 25% to 75% percentile; thin lines: minimal and maximal value,^0^: moderate outlier,. Mann Withney-U test. Quantitative realtime-PCR.

## Abbreviations

AUC: Area under the curve
Bak1: Bcl-2 antagonist killer1
BC: Breast cancer
cDNA: complementary DNA
DEPC: Diethylpyrocarbonate
HKG: Housekeeping gene
MDR1/P-glycoprotein: Multidrug resistance-1/P-glycoprotein
miR: miRNA, microRNA
MSU: Midstream specimens of urine
PDCD4: Programmed Cell Death 4
PTEN: Phosphatase and tensin homolog
qPCR: quantitative polymerase chain reaction
qRT-PCR: quantitative reverse transcriptase polymerase chain reaction
realtime-PCR: realtime polymerase chain reaction
RNA: Ribonucleic acid
ROC: Receiver Operating Characteristic
TPM1: Tropomyosin alpha-1 chain.
