# Supplementary figures and images for: Feasibility of urinary microRNA detection in breast cancer patients and its potential as an innovative non-invasive biomarker
Source: BMC Cancer. 2015 Mar 28;15:193. doi: 10.1186/s12885-015-1190-4 (PMC4383066; doi:10.1186/s12885-015-1190-4)

1

A

## Inter-assay variance

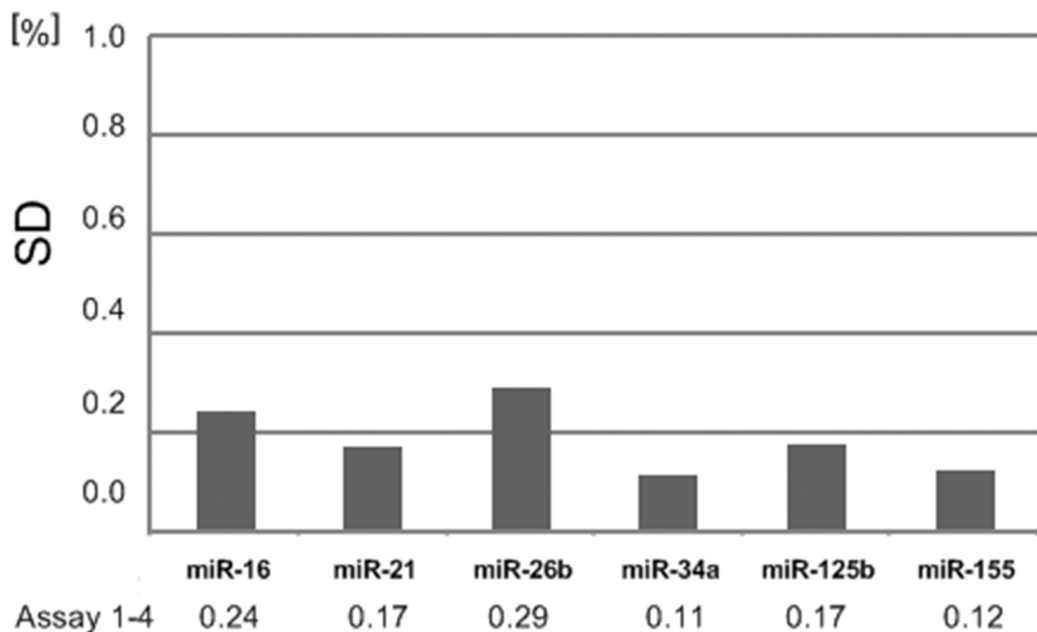

B

## Intra-assay variance

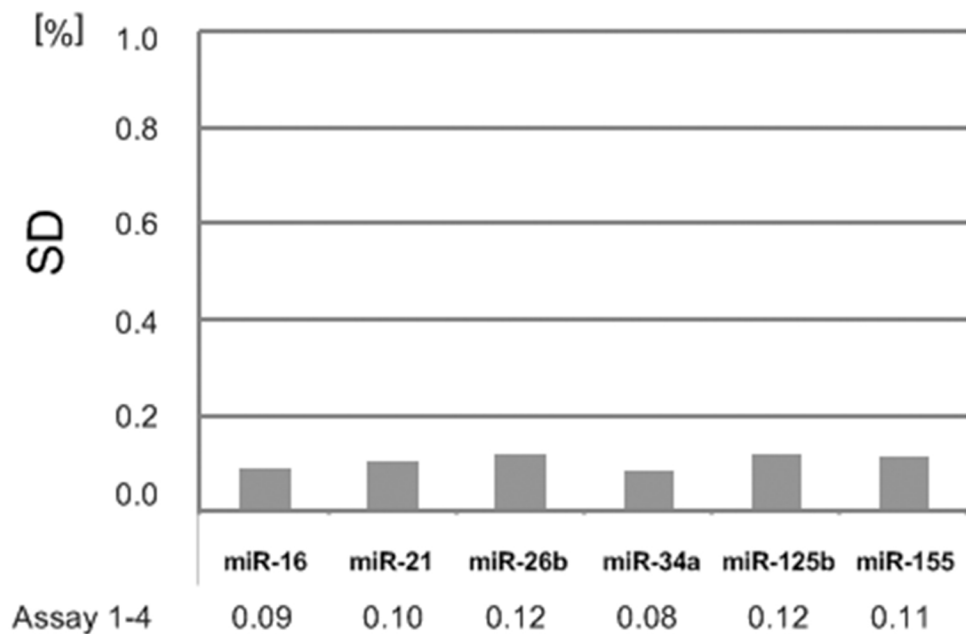

Supplement: Additional file 1: Figure S1. — Inter- and intra-assay variance in qPCR analysis in four assays. A. Inter-assay variance of miRNA types miR-16, −21, −26b, −34a, −125b, and −155. B. Intra-assay variance of miRNA types miR-16, −21, −26b, −34a, −125b, and −155, showing mean standard deviation (SD) in percentage [%] as vertical-bar diagram with numerical values below. [file 12885_2015_1190_MOESM1_ESM.pdf]

## HKG expression stability

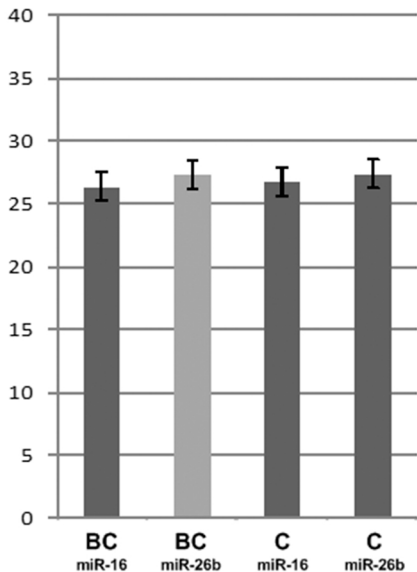

|          |       |       |       |       |
|----------|-------|-------|-------|-------|
| Geo mean | 26.34 | 27.28 | 26.72 | 27.37 |
| SD       | 2.26  | 2.38  | 2.13  | 1.57  |

Supplement: Additional file 2: Figure S2. — HKG expression stability in qPCR analysis. Expression values (geometric mean) of housekeeping miRNAs miR-16 and miR-26b in BC patients (BC) and healthy controls (C). Standard deviation (SD) and numerical values below vertical-bar diagram. [file 12885_2015_1190_MOESM2_ESM.pdf]

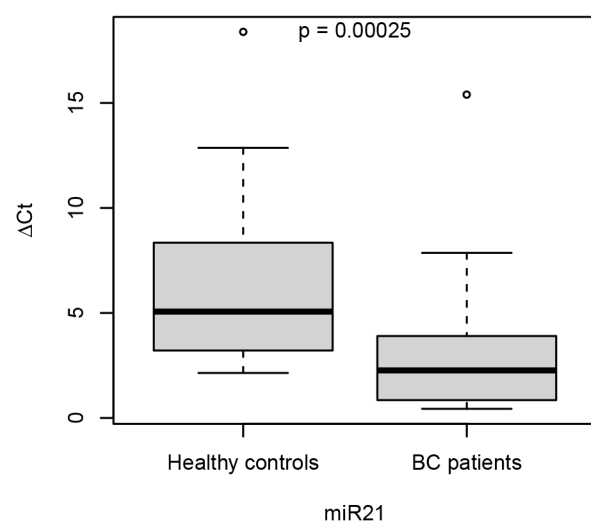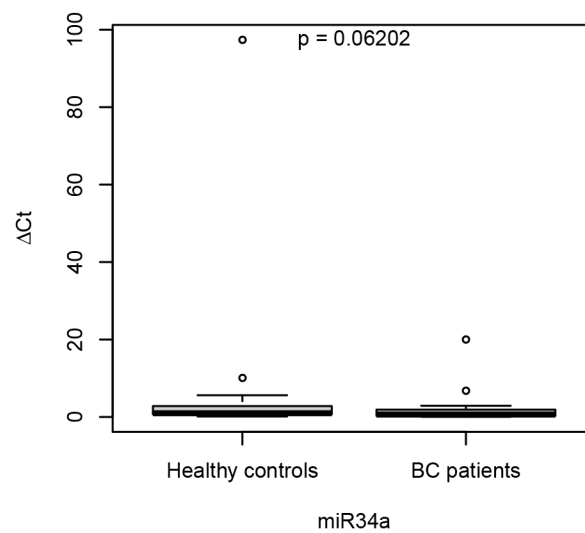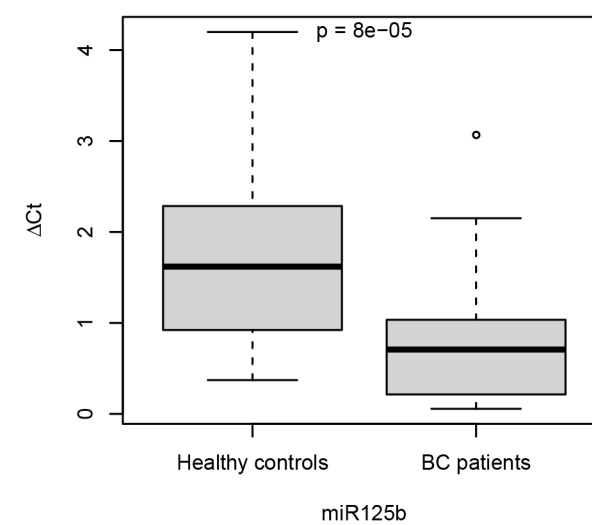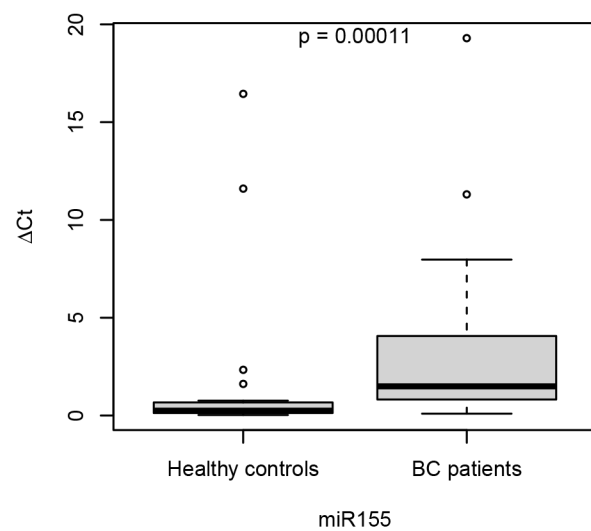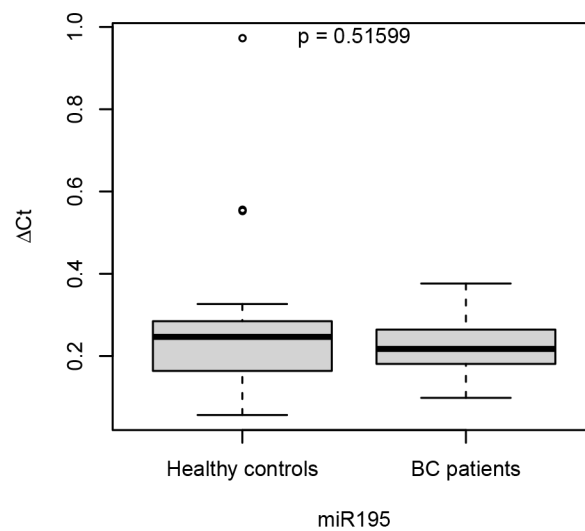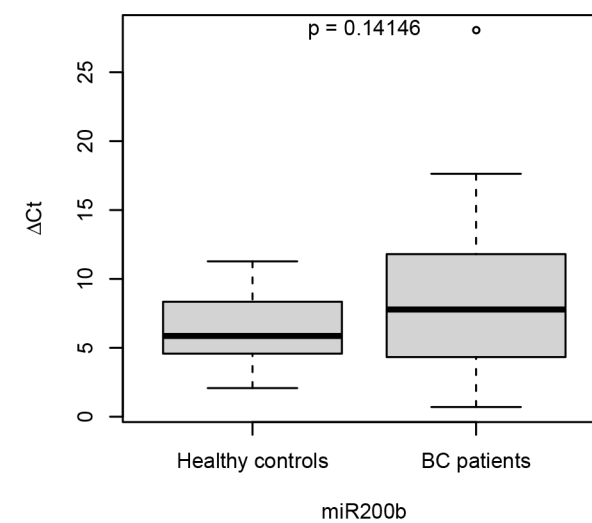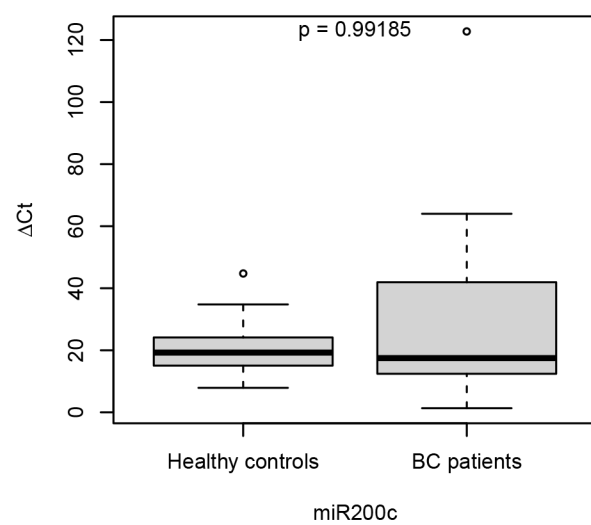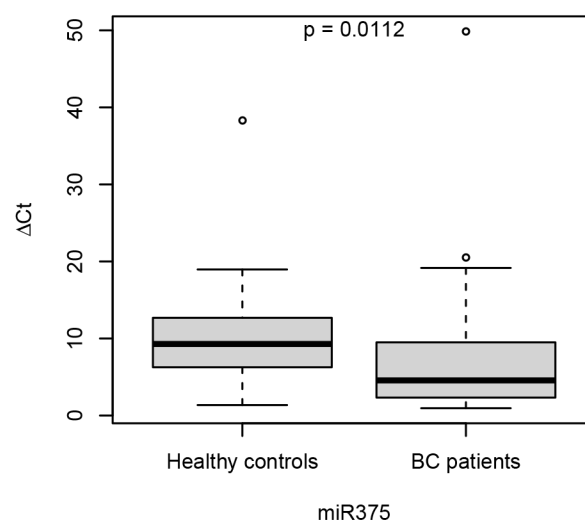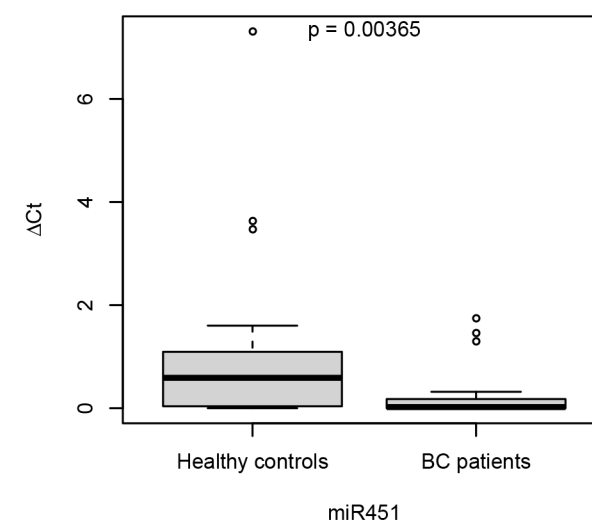

Supplement: Additional file 4: Figure S3. — Box plots of ΔCt-values of all nine investigated urinary miRNAs in breast cancer patients compared to healthy controls. Median urinary expression levels of miR-21, miR-34a, miR-125b, miR-155, miR-195, miR-200b, miR-200c, miR-375, and miR-451. Median ΔCt-value and interquartile range of duplicate experiments. Thick lines: median (50% percentile); gray boxes: 25% to 75% percentile; thin lines: minimal and maximal value,0: moderate outlier,. Mann Withney-U test. Quantitative realtime-PCR. [file 12885_2015_1190_MOESM4_ESM.pdf]
