# Supplementary material for: Feasibility of urinary microRNA detection in breast cancer patients and its potential as an innovative non-invasive biomarker
Source: BMC Cancer. 2015 Mar 28;15:193. doi: 10.1186/s12885-015-1190-4 (PMC4383066; doi:10.1186/s12885-015-1190-4)
Supplement: Additional file 3: Table S1. — Expression levels of urinary miRNAs of BC patients and healthy controls. Median urinary expression levels of nine breast cancer-related miRNAs in 24 BC patients and 24 healthy controls. Mann Withney-U test, interquartile range in parentheses.Table S2. Comparison of miRNA expression levels in serum of BC patients and controls. Table S3. Comparison of miRNA expression levels in serum and in urine of BC patients. Table S4. Comparison of miRNA expression levels in serum and in urine of controls. [file 12885_2015_1190_MOESM3_ESM.docx]

**Additional file 3**

**Table S1: Expression levels of urinary miRNAs of BC patients and healthy controls**

Median urinary expression levels of nine breast cancer-related miRNAs in 24 BC patients and 24 healthy controls. *Mann Withney-U test, interquartile range in parentheses.*

|  | **Median urine level in BC patients** | **Median urine level in healthy controls** | **p value** |
| --- | --- | --- | --- |
| **miR-21** | 2.27 (0.79-4.01) | 5.07 (3.15-8.51) | < 0.001 |
| **miR-34a** | 0.62 (0.29-2.03) | 0.97 (0.69-2.91) | 0.061 |
| **miR-125b** | 0.71 (0.21-1.07) | 1.62 (0.91-2.29) | <0.001 |
| **miR-155** | 1.49 (0.80-4.58) | 0.25 (0.12-0.71) | <0.001 |
| **miR-195** | 0.22 (0.18-0.27 ) | 0.25 (0.16-0.29) | 0.509 |
| **miR-200b** | 7.78 (4.24-12.20) | 5.86 (4.57-8.50) | 0.138 |
| **miR-200c** | 17.47 (12.38-44.07) | 19.26 (14.49-24.49) | 0.984 |
| **miR-375** | 4.56 (2.25-9.81) | 9.29 (6.24-12.80) | 0.011 |
| **miR-451** | 0.02 (0-0.22) | 0.59 (0.04-1.13) | 0.004 |

**Table S2:** Comparison of miRNA expression levels in serum of BC patients and controls

|  | Median serum level in BC patients | Median serum level  in healthy controls | p value |
| --- | --- | --- | --- |
| miR-21 | 0.037 (0.023-0.061) | 0.052 (0.043-0.070 | 0.248 |
| miR-34a | 0.0000 (0.0000-0.0007) | n.e. |  |
| miR-125b | 0.004 (0.0017-0.0047) | 0.0055 (0.004-0.011) | 0.189 |
| miR-155 | 0.0805 (0.0697-0.122) | 0.0910 (0.056-0.123) | 0.773 |
| miR-195 | 0.4820 (0.3492-0.4970) | 0.4525 (0.4385-0.4627) | 0.245 |
| miR-200b | 0.0010 (0.00025-0.0010) | 0.0010 (0.00025-0.0010) | 1.000 |
| miR-200c | 0.0050 (0.0035-0.0057) | 0.0045 (0.0040-0.0050) | 1.000 |
| miR-375 | n.e. |  |  |
| miR-451 | 14.704 (12.279-16.514 | 16.195 (11.986-18.881) | 0.564 |

**Table S3:** Comparison of miRNA expression levels in serum and in urine of BC patients

|  | Median serum level in BC patients | Median urine level in BC patients | p value |
| --- | --- | --- | --- |
| miR-21 | 0.037 (0.023-0.061) | 2.519 (2.206-3.735) | 0.021 |
| miR-34a | 0.0000 (0.0000-0.0007) | 0.4351 (0.2969-0.5947) | 0.018 |
| miR-125b | 0.004 (0.0017-0.0047) | 0.9104 (0.8781-0.9469) | 0.020 |
| miR-155 | 0.0805 (0.0697-0.122) | 0.8287 (0.5081-1.1731) | 0.021 |
| miR-195 | 0.4820 (0.3492-0.4970) | 0.1492 (0.1131-0.1825) | 0.020 |
| miR-200b | 0.0010 (0.00025-0.0010) | 5.8679 (3.9532-5.8679) | 0.018 |
| miR-200c | 0.0050 (0.0035-0.0057) | 12.3408 (8.1238-15.9909) | 0.020 |
| miR-375 | n.e. | 4.6812 (2.6637-7.9188) | 0.014 |
| mir-451 | 14.704 (12.279-16.514) | 0.0337 (0.0088-0.2125) | 0.021 |

**Table S4:** Comparison of miRNA expression levels in serum and in urine of controls

|  | Median serum level in healthy controls | Median urine level in healthy controls | P value |
| --- | --- | --- | --- |
| miR-21 | 0.052 (0.043-0.070) | 2.937 (2.3388-3.33379) | 0.021 |
| miR-34a | n.e. | 0.8803 (0.4586-1.0819) | 0.014 |
| miR-125b | 0.0055 (0.004-0.011) | 0.9221 (0.8903-1.1966) | 0.021 |
| miR-155 | 0.0910 (0.056-0.123) | 0.2819 (0.1109-0.6584) | 0.083 |
| miR-195 | 0.4525 (0.4385-0.4627) | 0.2515 (0.1786-0.2661) | 0.021 |
| miR-200b | 0.0010 (0.00025-0.0010) | 6.9889 (5.6739-7.7862) | 0.018 |
| miR-200c | 0.0045 (0.0040-0.0050) | 18.8527 (17.3459-21.2209) | 0.019 |
| miR-375 | n.e. | 11.1527 (6.6177-16.3017) | 0.014 |
| mir-451 | 16.195 (11.986-18.881) | 0.9886 (0.2645-1.0246) | 0.020 |
